# Comparison of ultrasonography and pathology features between children and adolescents with papillary thyroid carcinoma

**DOI:** 10.1016/j.heliyon.2023.e12828

**Published:** 2023-01-12

**Authors:** Yue Jie, Jingliang Ruan, Yuechang Cai, Man Luo, Rongbin Liu

**Affiliations:** a Department of Ultrasound, Sun Yat-Sen Memorial Hospital, Sun Yat-Sen University, Guangzhou, China; b Guangdong Provincial Key Laboratory of Malignant Tumor Epigenetics and Gene Regulation, Sun Yat-Sen Memorial Hospital Sun Yat-Sen University, Guangzhou, China

**Keywords:** Papillary thyroid carcinoma, Childhood, Adolescents, Ultrasonography, Molecular characteristics, PTC, Papillary thyroid cancer, ATA, American thyroid association, ACR, American College of Radiology, FNAC, Fine needle aspiration cytology, TI-RADS, Thyroid imaging reporting and data system, AJCC, American Joint Committee on Cancer, RET, Ret proto-oncogene, TRIM33, Tripartite motif-containing 33, CCDC6, Coiled-coil domain containing 6, NGS, Next-generation sequencing

## Abstract

**Objective:**

To compare the ultrasonography and pathology features between children and adolescents with papillary thyroid carcinoma (PTC).

**Methods:**

A total of 53 patients who were surgically diagnosed with childhood or adolescent PTC between 2017 and 2022 were included in this study. The pre-operative ultrasonography, post-operative histology, and molecular and clinical characteristics were retrospectively analyzed.

**Results:**

No differences were observed in composition, echogenicity, and shape using ultrasonography. Moreover, there was a significantly higher rate of extrathyroidal extension, punctate echogenic foci, and lymph node metastases in children compared to adolescents. The molecular analysis showed that BRAF^V600E^ mutations are the most prevalent abnormality in adolescent PTC (12/20, 60.0%). However, they are less in childhood PTC (7/23, 30.4%). In addition, using next-generation sequencing, three cases with oncogenic fusion (one TRIM33-RET case, one CCDC6-RET case, and one STRN-ALK case) were identified in childhood PTC.

**Conclusion:**

The frequency of extrathyroidal extension, punctate echogenic foci, and lymph node metastases were higher in childhood PTC, while BRAF^V600E^ mutations were higher in adolescent PTC.

## 1 Introduction

Papillary thyroid cancer (PTC) has been rarely reported in children and adolescents, accounting for 1.8–5.0% of all periods between malignancies [1,2] and 90% or more of all cases in children [3,4]. However, pediatric PTC typically has a better prognosis than PTC in adults despite tending to present at an advanced stage [[5], [6], [7]]. According to the American Thyroid Association (ATA) guidelines for pediatric thyroid nodules and cancer, the upper limit pediatrics should be regarded as those under 18 years of age to define the impact on tumor behavior more precisely [8]. According to previous studies, there are certain differences between the characteristics of children (≤15 years) and adolescents (>15 years), such as the 10-fold higher cancer incidence in adolescents between the age of 15–18 years [2,[9], [10], [11]]. Moreover, the female prevalence is also greater in this age group [12]. Above all, it is suggested that there are significant differences between childhood and adolescent PTC [[13], [14], [15], [16]].

Therefore, this retrospective study aimed to evaluate the differences between childhood and adolescent PTC in terms of pre-operative ultrasonography, pathological features, and molecular and clinical characteristics.

## 2 Materials and methods

### 2.1 Patients

The medical records of children and adolescents who underwent thyroidectomy and lymph node dissection for PTC between January 2017 and January 2022 were reviewed. 53 patients who met our inclusion criteria were included. The data for sex, age, pre-operative ultrasonography, post-operative histology, molecular, and clinical characteristics were collected. All surgeries were performed at our hospital. The study was approved by the Sun Yat-sen Memorial Hospital Sun Yat-sen University Institutional Review Board, and informed consent was obtained from the patients before the study.

### 2.2 Ultrasound imaging analysis

Ultrasound imaging was performed using a 7–11 MHz linear array transducer (iU22; Philips, Seattle, WA, USA), a 6–15 MHz linear array transducer (GE Logiq E9; GE Healthcare, Milwaukee, Wisconsin, USA), and a 5–13 MHz linear array transducer (Hi-Vision; Preirus, Hitachi, Tokyo, Japan).

Pre-operative ultrasound imaging was performed by a board-certified radiologist who specialized in ultrasound examinations and recorded ultrasound features and the category of each nodule in accordance with the American College of Radiology Thyroid Imaging Reporting and Data System (ACR TI-RADS) classification. Two board-certified senior radiologists, who were blind to the clinical data of all patients, reviewed the ultrasound images and reevaluated each nodule in terms of the primary tumor size, tumor location, quantity, with or without lymph node metastasis, ACR TI-RADS point scores, and specific ACR TI-RADS classification (TR1, TR2, TR3, TR4, and TR5). The final ACR TI-RADS point scores and specific ACR TI-RADS classification were discussed, taking the average as an agreement. The ACR TI-RADS classification concludes as follows: composition, echogenicity, shape, margin, and echogenic foci.

### 2.3 Pathological features analysis

Pathologic data, including tumor size, histologic classification, lymph node metastases, extrathyroidal extension, thyroiditis, Graves’ disease, calcifications, and BRAF^V600E^ mutation status, were obtained from clinical records. These pathologic data were further reviewed and analyzed. According to the American Joint Committee (AJCC) on Cancer eighth edition cancer staging manual for differentiated thyroid cancer (DTC) [17], the NM stage was evaluated considering tumor size, histologic classification, lymph node metastasis, and extrathyroidal extension.

Four children diagnosed with PTC underwent next-generation sequencing (NGS) with a panel of 88 thyroid carcinoma-related genes at Rigen Biotechnology (Rigen Biotechnology Co., Ltd, Shanghai, China) using the Nextseq500 sequencer (Illumina, San Diego, CA, USA). The genes included in the panel are listed in Supplementary T. 1. According to the manufacturer's instructions, 39 patients underwent BRAF^V600E^ mutation testing using an ARMS PCR kit (Amoy Diagnostics Co. Ltd, Xiamen, China), while the other eleven patients have no records of inquiries.

### 2.4 Statistical analysis

SPSS version 24.0 software (IBM Corporation, Armonk, NY) was used for performing the statistical analysis. The correlation analysis and paired *t*-test were utilized to evaluate a constant difference between two sets of values. All tests were 2-sided, and p < 0.05 was considered statistically significant with 95% confidence intervals (95% CI). The categorical variables were presented as numbers and percentages. Continuous variables were presented as mean ± standard deviation (SD).

## 3 Results

### 3.1 Patients’ characteristics

**Fig. 1:**
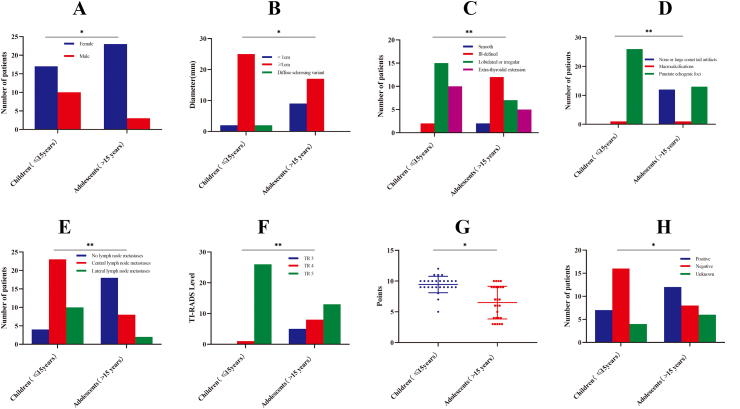
Significant comparison between childhood PTC and adolescent PTC.

In this cohort of 53 patients with PTC, there were 27 cases of children PTC (median age: 12 years, range: 3–15 years) and 26 cases of adolescent PTC (median age: 17 years, range: 16–18 years), including 40 (75.5%) females and 13 (24.5%) males. The differences in the characteristics of children (≤15 years) and adolescents (>15 years) with PTC are compared in Table 1. In this study, the female prevalence is higher in adolescent PTC compared to childhood PTC (Fig. 1A). The incidence of primary tumor size (≥1 cm) is also higher in adolescents (Fig. 1B). There were no significant differences in tumor multifocality, tumor location, the extent of thyroidectomy, and neck dissection between the two groups.

**Table 1:** Characteristics differentiation between children and adolescent patients with papillary thyroid carcinoma.

Characteristics	Children (≤15 years)	Adolescents (>15 years)	χ^2^	p
Sex				
Female	17	23	4.652	0.031
Male	10	3		
Multifocality				
Present	10	4	3.195	0.074
Absent	17	22		
Primary tumor size (cm)				
<1	2	9	5.962	0.015
≥1	25	17		
Diffuse sclerosing variant	2	0		
Tumor location				
Upper	8	6	0.293	0.589
Middle/lower	19	20		
Extent of thyroidectomy				
Total thyroidectomy (TT)	15	16	0.195	0.659
Less than TT	12	10		
Neck dissection				
Yes	24	20	1.345	0.246
No	3	6

### 3.2 Ultrasonographic features of children and adolescents with PTC

The ultrasonographic features of thyroid nodules in children (≤15 years) and adolescents (>15 years) were compared (Table 2). There were no differences in the composition (*P* = 0.304), echogenicity (*P* = 0.646), and shape (*P* = 0.322). However, there was a significantly higher rate of extrathyroidal extension [10/27 (37.0%) vs. 5/26 (19.2%), *P* = 0.003] (Fig. 1C), punctate echogenic foci [26/27 (96.3%) vs. 13/26 (50.0%), *P* = 0.001] (Fig. 1D), central/lateral lymph node metastases [23/27 (85.2%) vs. 8/26 (30.8%), *P* = 0.001] (Fig. 1E), TR5 lesions [26/27 (96.3%) vs. 13/26 (50.0%), *P* = 0.001] (Fig. 1F), and total ACR TI-RADS points (9.44 ± 1.34 vs. 6.50 ± 2.66, *P* = 0.001) (Fig. 1G) in the children group compared to the adolescent group.

**Table 2:** Ultrasonographic differentiation between children and adolescent patients with papillary thyroid carcinoma.

Ultrasonographic	Children (≤15 years)	Adolescents (>15 years)	χ^2^	P
Composition				
Mixed cystic and solid	0	1	1.0584	0.304
Solid or almost completely solid	27	25		
Echogenicity				
Hyperechoic or isoechoic	3	4	0.211	0.646
Hypoechoic	24	22		
Shape				
Wider-than-tall	26	26	0.981	0.322
Taller-than-wide	1	0		
Margin				
Smooth	0	2	13.705	0.003
Ill-defined	2	12		
Lobulated or irregular	15	7		
Extra-thyroidal extension	10	5		
Echogenic foci				
None or large comet tail artifacts	0	12	16.320	0.001
Macrocalcifications	1	1		
Punctate echogenic foci	26	13		
Lymph node metastases				
No lymph node metastases	4	18	16.154	0.001
Central lymph node metastases only	23	8		
Lateral lymph node metastases	10	2		
TI-RADS Level				
TR 3	0	5	14.764	0.001
TR 4	1	8		
TR 5	26	13		
Total points	9.44 ± 1.34	6.50 ± 2.66	3.933	0.001

### 3.3 Pathologic features of children and adolescents with PTC

A total of 43 (out of 53) patients were tested with BRAF^V600E^ mutations (Supplementary T. 2). The BRAF^V600E^ mutations were highly prevalent in adolescents with PTC (12/20, 60.0%) but less prevalent in children with PTC (7/23, 30.4%) (Table 3, Fig. 1H). However, there were no significant differences in the frequency of extrathyroidal extension/invasion, Hashimoto's thyroiditis, Graves' disease, psammoma bodies, pT staging, and pN staging between the two groups.

**Table 3:** Pathologic features differentiation between children and adolescent patients with papillary thyroid carcinoma.

Pathologic features	Children (≤15 years)	Adolescents (>15 years)	χ2	p
pT staging				
T1	9	9	0.246	0.884
T2	14	12		
T3	4	5		
pN staging				
N0	5	8	2.674	0.263
N1a	9	11		
N1b	13	7		
Extrathyroidal extension				
Yes	17	11	2.268	0.132
No	10	15		
Capsular invasion				
Yes	23	17	2.805	0.094
No	4	9		
Hashimoto's thyroiditis				
With	7	7	0.007	0.934
Without	20	19		
Graves' disease				
With	1	4	2.115	0.146
Without	26	22		
Psammoma bodies				
With	11	12	0.158	0.691
Without	16	14		
BRAF^V600E^ mutation				
Positive	7	12	4.385	0.036
Negative	16	8		
Unknown	4	6		
Other mutation	TRIM33-RET (T18:R12) CCDC6-RET (C1:R11)			
	STRN-ALK (S3:A20)			

**Fig. 2:**
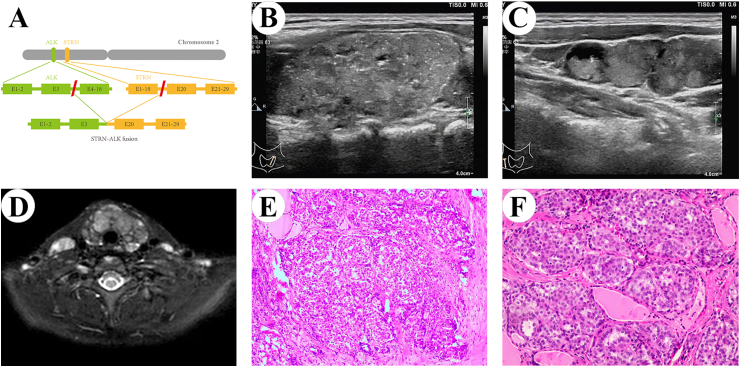
A rare STRN-ALK fusion in children PTC identified using next-generation sequencing. A. The fusion pattern of STRN-ALK; B. A longitudinal sonogram of the left lobe of the thyroid revealed an ill-defined hypoechoic mass with diffused and scattered microcalcification occupying the entire gland; C. Sonogram indicating level IV lymph node metastasis; D. Magnetic resonance (MR) images of the thyroid gland; E. Histopathological examination demonstrated multinodular growth at low magnification with traversing fibrosis; F. Follicular architecture was observed at high magnification.

Targeted next-generation sequencing using a panel of 88 thyroid carcinoma-related genes was carried out on fine needle aspiration cytology (FNAC) samples from four cases of childhood PTC (Supplementary T. 1 and 2). One case harbored BRAF^V600E^ mutations, while three cases (one tripartite motif motif-containing 33 (TRIM33) -ret proto-oncogene (RET) case, one coiled-coil domain containing 6 (CCDC6) -RET case, and one STRN-ALK case) with oncogenic fusion were identified (Fig. 2A). In this group, oncogenic fusion is commonly associated with more aggressive tumor behavior, including a large primary tumor size and a high incidence of lateral lymph node metastasis (Fig. 2B–F).

## 4 Discussion

Thyroid cancer in children and adolescents is increasing annually at a rate of 1.75/100,000 [1,18,19]. It is influenced by factors such as heredity, environment, hormones, etc. PTC is more invasive and prone to metastasis in children under 15 [20], including regional lymph node metastasis, extrathyroidal extension, and pulmonary metastasis. Furthermore, childhood PTC has a substantially lower mortality rate than adolescent PTC, with about 2% or less long-term cause-specific mortality [8].

Childhood PTC may progress to a higher pTNM stage and have a higher frequency of microcalcification in nodules on ultrasonography [21]. In ACR TI-RADS classifications, childhood PTC received more point scores, which resulted in a higher category. Therefore, pediatric thyroid cancer should be considered when a child's thyroid nodule is observed on ultrasound and appears solid and grows rapidly, especially with calcification [22,23]. Furthermore, when patients suffer from PTC, cervical lymph node metastasis should be highly suspected [24]. The sonographer can directly recommend the patient to get an FNAC examination done if there is a highly suspicious thyroid nodule or enlarged lymph node [25]. The FNAC examination result is the basis for the clinician to make a surgical decision [26].

The findings revealed that childhood PTC had more lymphatic metastasis and microcalcification than adolescent PTC, which resulted in higher ACR TI-RADS points and was associated with a higher tumor stage [27]. Moreover, the results indicate that PTC in these two age groups may have different pathogenesis. According to a previous study, we tended to classify juvenile (age ≤18 years) PTC as a disease. However, childhood PTC revealed larger tumor size, increased lymphatic metastasis, and higher pulmonary metastasis rate [28]. According to the 2015 ATA Management Guidelines for Children with Thyroid Nodules and Differentiated Thyroid Cancer, the American Thyroid Association Guidelines Task Force on Pediatric Thyroid Cancer, childhood PTC is divided into low-risk, intermediate-risk, and high-risk categories based on clinical manifestations, tumor size, regional invasion, and metastasis [3].

The incidence of female patients in adolescent PTC is higher than male patients [29]. The reason for the gender difference in incidence is still not clear. According to some studies, there is a gender difference in incidence starting from the age of 15 [29]. Moreover, some studies have revealed that such sex differences may be associated with high estrogen levels, which may be crucial to this process [30,31]. Other studies suggest that the significant physiologic changes associated with different life stages affect the timing of the presentation of thyroid disease in women. The body's demand for thyroid hormones increases during growth, and its interaction with sex hormones may result in the development of thyroid nodules. Thyroid cancer is more likely to develop when the serum levels of estrogen and progesterone are higher. Additionally, negative emotions may elevate the secretion of adrenal corticosteroids, causing immune system dysfunction and resulting in malignant tumors [32]. As our study is a retrospective analysis, the sample size has certain limitations. There could be some statistical deviation because there were only three adolescent male patients in the study.

Although BRAF^V600E^ mutations are not common in childhood PTC, their mutation tends to increase with aging [33]. A previous study has demonstrated that the incidence of BRAF^V600E^ mutation in pediatric PTC is substantially lower than in adult PTC (20% vs. 77%) [34]. In the present study, 43 patients (out of 53) were tested with BRAF^V600E^ mutations (Supplementary T. 2). It indicates that the BRAF^V600E^ mutation rate was lower in children than in adolescents with PTC (30.4% vs. 60%, *P* = 0.036). Clinically, unlike PTC in adults, BRAF^V600E^ mutation-associated tumors in children do not indicate a more aggressive biological behavior [35]. RET/PTC rearrangement is the most common genetic alteration identified in PTC. Approximately 10–20% of the tumors have RET/PTC rearrangement, in which the most common fusion partners are CCDC6-RET and RCON4-RET. RET/PTC rearrangement rate in children is much higher than the mutation rate in adults (47–65% vs. 3–34%) [36]. Additionally, in childhood PTC, the presence of RET/PTC rearrangement is associated with extracapsular invasion, lymph node metastases, and distant metastases appearing earlier, with higher staging and histology grades [37,38]. In summary, the proportion of BRAF^V600E^ gene mutation in childhood PTC is lower than in adults, and RET-fusion is frequently found in children.

To summarize, there were some differences in ultrasonography and pathological features between childhood PTC and adolescents PTC. Childhood PTC had a higher risk of extrathyroidal extension and lymph node metastasis. RET/PTC rearrangement rate was higher, but BRAF^V600E^ mutations were less in childhood PTC. The BRAF^V600E^ mutations were used as an evaluation index for the risk stratification of adult thyroid cancer. Since the BRAF^V600E^ mutations differed between childhood PTC and adolescents PTC, the risk stratification should be integrated with ultrasonography, clinical symptoms, tumor size, post-surgery serum thyroglobulin level, etc. Due to the high invasiveness of childhood PTC, surgical techniques, including total thyroidectomy or thyroid lobectomy, whether they have peripheral lymph node dissection, need to be comprehensively evaluated. The collaborative diagnosis and treatment of multidisciplinary teams are crucial. It could guarantee the precise treatment of thyroid cancer, thus improving the prognosis.

Since it is a retrospective study, there are certain limitations. Not all patients underwent the BRAF^V600E^ mutation testing. Some children in our study had a multifocal disease found after surgery. The follow-up data, like whether have recurrence or not, is not concrete. The insufficient number of cases in our study is another potential limitation. Therefore, large-sample, multicenter prospective studies are needed to investigate the differences between children and adolescents with PTC.

In conclusion, childhood PTC had a higher frequency of extrathyroidal extension, punctate echogenic foci, and lymph node metastases than adolescent PTC. Furthermore, BRAF^V600E^ mutations were high in adolescent PTC and adolescent PTC was found to be more prevalent in females, which may be linked to elevated estrogen levels.

## Appendix A Supplementary data

Supplementary_files__spl_2_spl_ Supplementary_files__spl_2_spl_
