# Supplementary material for: Comparison of ultrasonography and pathology features between children and adolescents with papillary thyroid carcinoma
Source: Heliyon. 2023 Jan 12;9(1):e12828. doi: 10.1016/j.heliyon.2023.e12828 (PMC9871215; doi:10.1016/j.heliyon.2023.e12828)
Supplement: Supplementary_files__spl_2_spl_ [file mmc1.docx]

Supplementary Table 1. Next-generation sequencing (NGS) technology was used to detect 88 thyroid cancer-related genes, and the detection range included the full exon coverage of 86 genes and 6 gene rearrangements. The 88 genes in the panel are as follows:

| AAAS | BRAF | CTNNB1 | ERCC1 | HRAS | MET | NRAS | PTPN11 | SLC15A2 | TP53 |
| --- | --- | --- | --- | --- | --- | --- | --- | --- | --- |
| ABCB1 | CDH1 | CYP1B1 | ERCC2 | IDH1 | METTL14 | NRG3 | RAB42 | SLC26A3 | TSHR |
| AKT1 | CDK5RAP2 | DDX3X | ETV6 | JAK2 | MLH1 | NTRK1 | RANBP17 | SMAD4 | VHL |
| ALK | CDKN2A | DICER1 | FAM81B | KDM6B | MRPL50 | OR52D1 | RB1 | SMARCA4 | XRCC1 |
| ALMS1 | CDKN2C | DISP2 | FAT4 | KRAS | MSH2 | OVCH1 | RELN | SPTBN5 | ZAN |
| APC | CHEK1 | DOCK9 | FBXW7 | KRTAP10-5 | MSH6 | PIK3CA | RET | STK11 | ZNF292 |
| ASPM | CHEK2 | EGFR | GABRA2 | LPA | MYH7 | PPARG | SEMA6A | TDG | ZNF878 |
| ATM | CHGA | EIF1AX | GNAS | MDC1 | NF1 | PPM1D | SF3B1 | TERT |  |
| AXIN1 | CPAMD8 | EPCAM | GSTP1 | MEN1 | NF2 | PTEN | SH3BP5 | TIRAP |  |

Point mutation Gene rearrangements Point mutation &Gene rearrangements

| Supplementary Table 2. BRAFV600E mutation status of 53 panties according to pathological results | | | | | | |
| --- | --- | --- | --- | --- | --- | --- |
| **Case** | **Sex** | **Age** | **Stage（AJCC）** | **BRAF Mutation** | **Detection method** | **Histological Type** |
| 1 | Male | 4 | 1 | Negative | PCR | PTC |
| 2 | Female | 8 | 1 | Negative | PCR | PTC |
| 3 | Female | 8 | 1 | Negative | NGS | PTC |
| 4 | Female | 9 | 1 | Positive | PCR | PTC |
| 5 | Male | 9 | 1 | Negative | PCR | PTC |
| 6 | Female | 10 | 1 | Negative | PCR | PTC |
| 7 | Female | 11 | 1 | N/A | PCR | PTC |
| 8 | Male | 11 | 1 | Positive | PCR | PTC |
| 9 | Female | 12 | 1 | Positive | NGS | PTC |
| 10 | Female | 12 | 1 | Negative | NGS | PTC |
| 11 | Female | 12 | 1 | Negative | PCR | PTC |
| 12 | Female | 12 | 1 | Negative | PCR | PTC |
| 13 | Female | 13 | 1 | Negative | PCR | PTMC |
| 14 | Female | 13 | 1 | Positive | PCR | PTMC |
| 15 | Female | 13 | 1 | N/A | PCR | PTC |
| 16 | Female | 14 | 1 | N/A | PCR | PTC |
| 17 | Female | 14 | 1 | Negative | PCR | PTC |
| 18 | Female | 14 | 1 | Negative | PCR | PTC |
| 19 | Male | 14 | 1 | Positive | PCR | PTMC |
| 20 | Male | 14 | 1 | N/A | PCR | PTC |
| 21 | Female | 14 | 1 | Negative | PCR | WT-UMP |
| 22 | Female | 14 | 1 | Negative | PCR | PTC |
| 23 | Female | 15 | 1 | Positive | PCR | PTC |
| 24 | Male | 15 | 1 | Negative | PCR | PTC |
| 25 | Male | 15 | 1 | Positive | PCR | PTC |
| 26 | Female | 15 | 1 | Negative | NGS | PTC |
| 27 | Female | 15 | 1 | Negative | PCR | PTC |
| 33 | Female | 16 | 1 | Positive | PCR | PTMC |
| 31 | Female | 16 | 1 | Negative | PCR | PTC |
| 32 | Female | 16 | 1 | Positive | PCR | PTC |
| 28 | Male | 16 | 1 | Negative | PCR | PTC |
| 34 | Female | 16 | 1 | Negative | PCR | PTC |
| 29 | Female | 16 | 1 | N/A | PCR | PTC |
| 30 | Female | 16 | 1 | N/A | PCR | PTC |
| 42 | Female | 17 | 1 | Positive | PCR | PTMC |
| 35 | Female | 17 | 1 | N/A | PCR | PTMC |
| 47 | Female | 17 | 1 | Positive | PCR | PTC |
| 46 | Male | 17 | 1 | Positive | PCR | PTMC |
| 36 | Female | 17 | 1 | Negative | PCR | PTC |
| 41 | Female | 17 | 1 | Positive | PCR | PTC |
| 38 | Female | 17 | 1 | N/A | PCR | PTC |
| 37 | Female | 17 | 1 | Positive | PCR | PTC |
| 40 | Female | 17 | 1 | Negative | PCR | PTC |
| 39 | Female | 17 | 1 | Negative | PCR | PTC |
| 43 | Female | 17 | 1 | Positive | PCR | PTC |
| 45 | Male | 17 | 1 | Positive | PCR | PTC |
| 44 | Female | 17 | 1 | Negative | PCR | PTC |
| 52 | Female | 18 | 1 | Positive | PCR | PTMC |
| 48 | Female | 18 | 1 | N/A | PCR | PTC |
| 50 | Female | 18 | 1 | Positive | PCR | PTC |
| 51 | Female | 18 | 1 | Positive | PCR | PTC |
| 49 | Female | 18 | 1 | N/A | PCR | PTC |
| 53 | Female | 18 | 1 | Negative | PCR | PTC |
| Abbreviations: BRAF, B-type Raf kinase, PTC, papillary thyroid carcinoma, PTMC, papillary thyroid microcarcinoma, WT-UMP, well differentiated tumors of uncertain malignant potential. | | | | | | |

Supplementary Table 3. Race and Surgery Methods of 53 patients

| Case | Sex | Age | Race | Surgery Method |  |
| --- | --- | --- | --- | --- | --- |
| 1 | M | 4 | Chinese | lobectomy of right thyroid | |
| 2 | F | 8 | Chinese | lobectomy of right thyroid | |
| 3 | F | 8 | Chinese | lobectomy of right thyroid | |
| 4 | M | 9 | Chinese | lobectomy of left thyroid | |
| 5 | F | 9 | Chinese | Lobectomy of right thyroid and subtotal lobectomy of left thyroid | |
| 6 | F | 10 | Chinese | lobectomy of left thyroid | |
| 7 | F | 11 | Chinese | total thyroidectomy | |
| 8 | M | 11 | Chinese | lobectomy of left thyroid | |
| 9 | F | 12 | Chinese | total thyroidectomy | |
| 10 | F | 12 | Chinese | lobectomy of left thyroid | |
| 11 | F | 12 | Chinese | lobectomy of right thyroid | |
| 12 | F | 12 | Chinese | Lobectomy of left thyroid and subtotal lobectomy of right thyroid | |
| 13 | F | 13 | Chinese | lobectomy of left thyroid | |
| 14 | F | 13 | Chinese | Lobectomy of right thyroid and subtotal lobectomy of left thyroid | |
| 15 | F | 13 | Chinese | lobectomy of right thyroid | |
| 16 | F | 14 | Chinese | total thyroidectomy | |
| 17 | F | 14 | Chinese | total thyroidectomy | |
| 18 | M | 14 | Chinese | total thyroidectomy | |
| 19 | F | 14 | Chinese | total thyroidectomy | |
| 20 | F | 14 | Chinese | total thyroidectomy | |
| 21 | F | 14 | Chinese | total thyroidectomy | |
| 22 | M | 14 | Chinese | total thyroidectomy | |
| 23 | M | 15 | Chinese | total thyroidectomy | |
| 24 | F | 15 | Chinese | lobectomy of right thyroid | |
| 25 | F | 15 | Chinese | Lobectomy of left thyroid and subtotal lobectomy of right thyroid | |
| 26 | F | 15 | Chinese | Lobectomy of right thyroid and subtotal lobectomy of left thyroid | |
| 27 | M | 15 | Chinese | total thyroidectomy | |
| 28 | M | 16 | Chinese | total thyroidectomy | |
| 29 | F | 16 | Chinese | lobectomy of left thyroid | |
| 30 | F | 16 | Chinese | lobectomy of left thyroid | |
| 31 | F | 16 | Chinese | subtotal thyroidectomy | |
| 32 | F | 17 | Chinese | lobectomy of right thyroid | |
| 33 | F | 17 | Chinese | total thyroidectomy | |
| 34 | F | 17 | Chinese | total thyroidectomy | |
| 35 | F | 17 | Chinese | lobectomy of right thyroid | |
| 36 | F | 17 | Chinese | lobectomy of left thyroid | |
| 37 | F | 17 | Chinese | total thyroidectomy | |
| 38 | F | 17 | Chinese | lobectomy of right thyroid | |
| 39 | F | 17 | Chinese | total thyroidectomy | |
| 40 | F | 18 | Chinese | total thyroidectomy | |
| 41 | F | 18 | Chinese | total thyroidectomy | |
| 42 | F | 18 | Chinese | total thyroidectomy | |
| 43 | F | 18 | Chinese | lobectomy of right thyroid | |
| 44 | F | 18 | Chinese | lobectomy of right thyroid | |
| 45 | F | 18 | Chinese | total thyroidectomy | |
| 46 | F | 18 | Chinese | lobectomy of right thyroid | |
| 47 | F | 18 | Chinese | total thyroidectomy | |
| 48 | F | 18 | Chinese | total thyroidectomy | |
| 49 | F | 18 | Chinese | total thyroidectomy | |
| 50 | F | 18 | Chinese | total thyroidectomy | |
| 51 | F | 18 | Chinese | total thyroidectomy | |
| 52 | F | 18 | Chinese | lobectomy of right thyroid | |
| 53 | F | 18 | Chinese | lobectomy of left thyroid | |
